# Perioperative and Survival Outcomes of Robotic-Assisted Surgery, Comparison with Laparoscopy and Laparotomy, for Ovarian Cancer: A Network Meta-Analysis

**DOI:** 10.1155/2022/2084774

**Published:** 2022-04-30

**Authors:** Qin Tang, Weichu Liu, Dan Jiang, Junying Tang, Qin Zhou, Jing Zhang

**Affiliations:** ^1^Department of Obstetrics and Gynecology, The First Affiliated Hospital of Chongqing Medical University, Chongqing 400016, China; ^2^Sichuan Provincial Maternity and Child Health Care Hospital, Chengdu, 610000 Sichuan, China

## Abstract

**Objective:**

We aimed to compare the perioperative and survival outcomes of robotic-assisted surgery, traditional laparoscopy, and laparotomy approaches in ovarian cancer.

**Methods:**

PubMed, Cochrane Library, Embase, Web of Science, and Chinese National Knowledge Infrastructure (CNKI) were searched using multiple terms for ovarian cancer surgeries, including comparative studies in Chinese and English. Literatures are published before August 31, 2021. The outcomes include operating time, estimated blood loss, length of hospital stay, postoperative/intraoperative/total complications, pelvic/para-aortic/total lymph nodes, transfusion, and five-year overall survival rate. The dichotomous data, continuous data, and OS data were pooled and reported as relative risk, standardized mean differences, and hazard ratio HRs with 95% confidence intervals, respectively. The Newcastle–Ottawa Scale was used to evaluate the risk of bias of included studies.

**Results:**

Thirty-eight studies, including 8,367 patients and three different surgical approaches (robotic-assisted laparoscopy surgery, traditional laparoscopy, or laparotomy approaches), were included in this network meta-analysis. Our analysis shows that the operating time of laparotomy was shorter than laparoscopy. The robotic-assisted laparoscopy has the least estimated blood loss during the surgery, followed by laparoscopy, and finally laparotomy. Compared with laparotomy, the incidence of blood transfusion was lower in the robotic-assisted laparoscopy and laparoscopy groups, and the length of hospital stay is shorter. Laparotomy had a significantly higher incidence of total complications than robotic-assisted laparoscopy and laparoscopy and higher postoperative complications than laparoscopy. For the number of pelvic/para-aortic/total lymph nodes removed by different surgical approaches, our analysis revealed no statistical difference. Our analysis also revealed no significant differences in intraoperative complications and 5-year OS among the three surgical approaches.

**Conclusion:**

Compared with laparotomy, robotic-assisted laparoscopy and laparoscopy had a shorter hospital stay, decreased blood loss, fewer complications, and transfusion happened. The 5-year OS of ovarian cancer patients has no difference between robotic-assisted laparoscopy, laparoscopy, and laparotomy groups.

## 1. Background

Ovarian cancer is one of the most common gynecological malignancies worldwide, with approximately 314,000 new cases and 207,000 deaths per year [1]. Because of the absence of clinical symptoms, more than two-thirds of the diagnoses are made at advanced stages, resulting in a poor 5-year survival rate, especially in epithelial ovarian cancer (EOC) [2]. The mainstay treatment of ovarian cancer is still the traditional radical surgery combined with platinum-based chemotherapy. Satisfactory cytoreductive surgery is beneficial for the prognosis of patients with advanced ovarian cancer [3].

Traditionally, the radical surgery of ovarian cancer has been performed via laparotomy with a longitudinal median incision. A recent multicenter retrospective review of long-term outcomes after staging minimally invasive surgery for early-stage ovarian cancer suggests that minimally invasive surgery is a valuable treatment option, but the patient needs to be selected appropriately [4]. Since the da Vinci robotic surgical system was cleared for use in gynecologic surgery in the USA in 2005, its application has rapidly become more comprehensive and widespread [5]. A robotic-assisted surgical system can provide instruments with a wrist function at the tip and a 360-degree range of motion, tremor filtration, a stable 3-dimensional vision, and an ergonomic working position [6]. It has been shown to be practical and feasible for staging and treating endometrial and cervical cancer [7, 8], whereas robotic-assisted laparoscopy surgery (RAS) in primary and recurrent ovarian cancers still remains an area of active study and debate. Recently, several meta-analyses [9–11] have directly compared the feasibility and safety between RAS, traditional laparoscopy (LS), or laparotomy (LT), but there is little literature about RAS. Therefore, we conducted a network meta-analysis, including more literature, to direct and indirect compare the efficacy and outcomes among RAS, LS, and LT in the treatment of ovarian cancer.

## 2. Materials and Methods

This network meta-analysis was carried out in accordance with the extension of the Preferred Reporting Items for Systematic Reviews and Meta-analyses (PRISMA) statement for Network Meta-analyses [12].

### 2.1. Data Sources and Search Strategy

PubMed, Cochrane library, Embase, Web of Science, and Chinese National Knowledge Infrastructure (CNKI) were systematically searched. The search terms were ovarian neoplasm, ovarian cancer, ovarian carcinoma, ovarian tumor, peritoneoscopy, celioscopy, laparoscope, endoscope, laparotomy, open surgery, robot-assisted surgery, robot surgery, robot-enhanced procedures, and robotic surgical procedure. Literatures published before August 31, 2021, were searched. Taking PubMed as an example, the specific search strategy is shown in Table 1.

### 2.2. Inclusion and Exclusion Criteria

The literature screening was performed by two investigators separately, and the disagreements were settled by discussing with the third investigator. The literature was selected with the following criteria: [1] patients diagnosed with ovarian cancer; [2] patients underwent radical surgery, which consists of surgical staging based on hysterectomy, bilateral adnexectomy, omentectomy, pelvic and aortic lymphadenectomy (or not), and multiple peritoneal biopsies, as well as appendectomy (for mucinous histology); [3] the studies compared the outcomes of robot-assisted surgery, laparoscopy, or laparotomy; the outcomes include five-year overall survival (OS) rate, estimated blood loss (EBL)/ml, length of hospital stay (LHS)/days, operating time (OT)/min, postoperative/intraoperative complications, and pelvic/para-aortic/total lymph nodes or include at least one of them; [4] the patients with or without neoadjuvant chemotherapy were all included; and [5] published English or Chinese literature was included. Meanwhile, the literature with the following criteria were excluded: [1] data were incomplete or could not be used for statistical analysis; [2] duplicate publications; studies were reviews, abstracts, letters, and comments; [3] non-English or non-Chinese language literature; and [4] studies with less than ten patients and studies including patients treated for recurrent ovarian cancer or fertility-sparing surgery only. References of the included papers were further searched to identify other potentially relevant studies.

### 2.3. Data Extraction and Quality Evaluation

The data extraction and quality evaluation were carried out by two investigators, respectively, and the disagreements were settled by discussing with the third investigator. The data extracted by a standard excel form including first author's name, year of publication, study time, location, stage of ovarian cancer, the number of patients, mean age, body mass index (BMI), study design, bias score, follow-up time, and the outcomes (including OT, EBL, LHS, postoperative/intraoperative/total complications, pelvic/para-aortic/total lymph nodes, transfusion, and five-years OS). Data presented as median values and ranges were converted to mean values and standard deviations (mean ± SD) using the formula proposed by Hayduk et al. [13]. For survival data, we extracted hazard ratio (HR) with a 95% confidence interval (CI) from included studies. If HR and 95% CI were not directly reported, we extracted data from Kaplan-Meier curves by Engauge Digitizeit 4.1, and we would calculate HR and 95% CI as described by Tierney [14].

We used the Newcastle–Ottawa Scale (NOS), which contained three components (selection, comparability, and outcome), to evaluate the risk of bias of included studies.

### 2.4. Statistical Analysis

Analyses were performed using Stata 14.0 (StataCorp, College Station, TX) and the R 4.0.3 software (R Foundation for Statistical Computing, Beijing, China, “meta” and “netmeata” and “gemtc” packages). For dichotomous and continuous data, we used frequentist method random-effects networks in this meta-analysis. The dichotomous data results were pooled and reported as relative risk (RRs) with 95% confidence intervals (CIs). The continuous data results were reported as standardized mean differences (SMDs) with 95% CIs. The data of OS was pooled using hazard ratio (HRs) and corresponding 95% CI. When there is a closed-loop, the consistency test is conducted between the direct comparison and the indirect comparison. When the inconsistency factor (IF) of the consistency test is close to 0, the direct and indirect evidence was considered to have consistency. Consistency between the direct and indirect evidence was also assessed by comparing the individual data point's posterior mean deviance contributions for the consistency and inconsistency model and node splitting analysis.

## 3. Results

### 3.1. Characteristics of Included Studies

**Figure 1:**
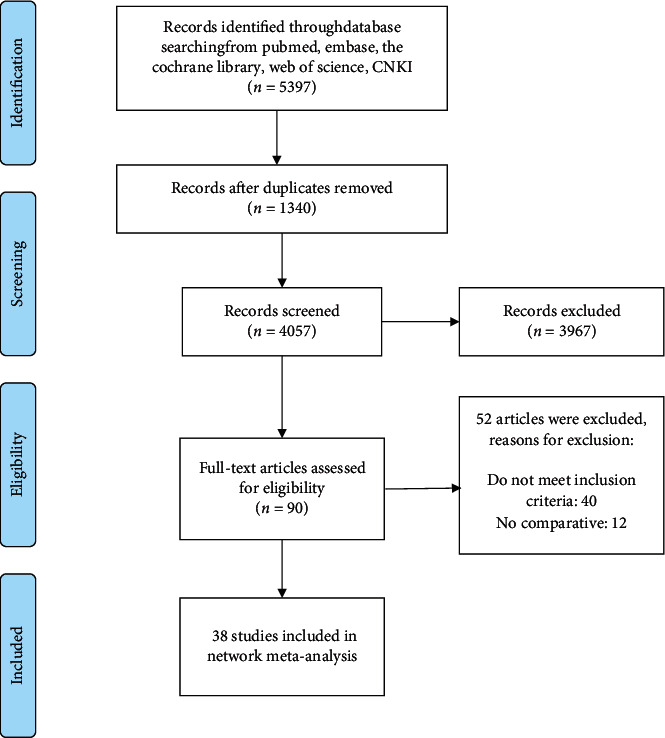
Flow diagram of study selection.

38 studies were included in the analysis, published from 2005 to 2020, and a total of 8367 women with ovarian cancer were enrolled. The study of Nezhat et al. (2014) [15] reported perioperative outcomes for stage I and II-IV ovarian cancer, respectively. Thus, we consider it as two studies. There were 6 three-arm studies comparing the perioperative and/or survival outcomes of ovarian cancer patients treated by robotic-assisted surgery, traditional laparoscopy, and laparotomy. There were 33 dual-arm studies, 27 of which compared laparoscopy and laparotomy, 4 compared robotic-assisted surgery and traditional laparoscopy surgery, and 2 compared robotic-assisted surgery and laparotomy. RAS-LS-LT was the only closed-loop included in the study. The characteristics of the included studies are shown in Table 2. The study selection flowchart (PRISMA) is shown in Figure 1.

### 3.2. Network Map

**Figure 2:**
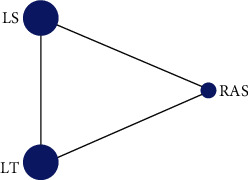
Network map of operating time.

The line between two nodes represents a direct comparison. The thicker the line, the more research. The larger the node, the larger the sample size. Since only three interventions were compared in this network meta-analysis, only one closed loop was formed. The network map for each outcome variable differs only in nodes size and line thickness. We only show the network map of OT (Figure 2), which with the largest number of research included.

### 3.3. Operating Time (OT)

**Figure 3:**
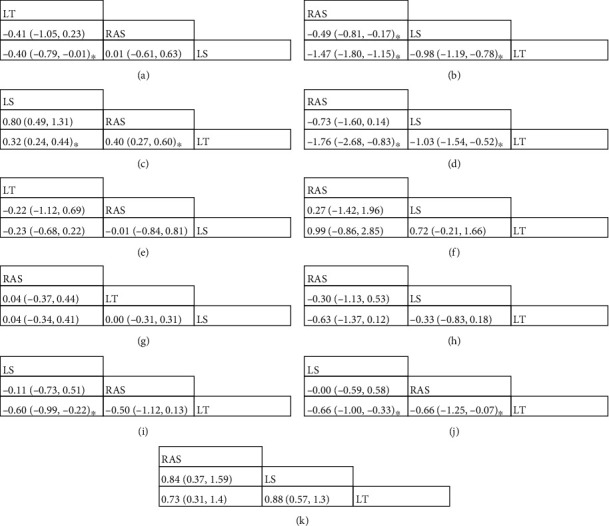
Network meta-analysis of perioperative outcomes Note: (a) operating time/min; (b) estimated blood loss (EBL)/ml; (c) transfusion; (d) length of hospital stay (LHS)/days; (e) pelvic lymph nodes; (f) para-aortic lymph nodes; (g) total lymph nodes; (h) intraoperative complications; (i) postoperative complications; (j) total complications; and (k) five-year overall survival (OS) rate. ∗*P* < .05.

31 studies reported the operating time of different surgical approaches. Our study shows that the OT was the shortest for LT followed by RAS and finally LS; results are shown in Figure 3(a). The comparison between LT and LS was statistically significant (*p* = .02). There was no significant difference between RAS and LS groups and RAS and LT groups (*p* > .05).

### 3.4. Estimated Blood Loss (EBL)

28 studies reported the estimated blood loss during surgery by different surgical approaches. Our study shows that the EBL was the lowest for RAS followed by LS and finally LT; results are shown in Figure 3(b). The comparisons between RAS and LT (*p* < .001), LS and LT (*p* < .001), and RAS and LS (*p* = .018) were statistically significant.

### 3.5. Transfusion

17 studies reported the incidence of transfusion with different surgical approaches. Statistical results show that the incidence of transfusion was the lowest for LS followed by RAS and finally LT; the results are shown in Figure 3(c). And the comparisons between LS and LT (*p* < .001) and RAS and LT (*p* = .004) were statistically significant.

### 3.6. Length of Hospital Stay (LHS)

26 studies reported the LHS (days) after surgery by different surgical approaches. Our study showed that the length of hospital stay was the shortest for RAS followed by LS and finally LT. The comparisons between RAS and LT and LS and LT are statistically significant (*p* < .001); results are shown in Figure 3(d).

### 3.7. Pelvic/Para-aortic/Total Lymph Nodes

**Figure 4:**
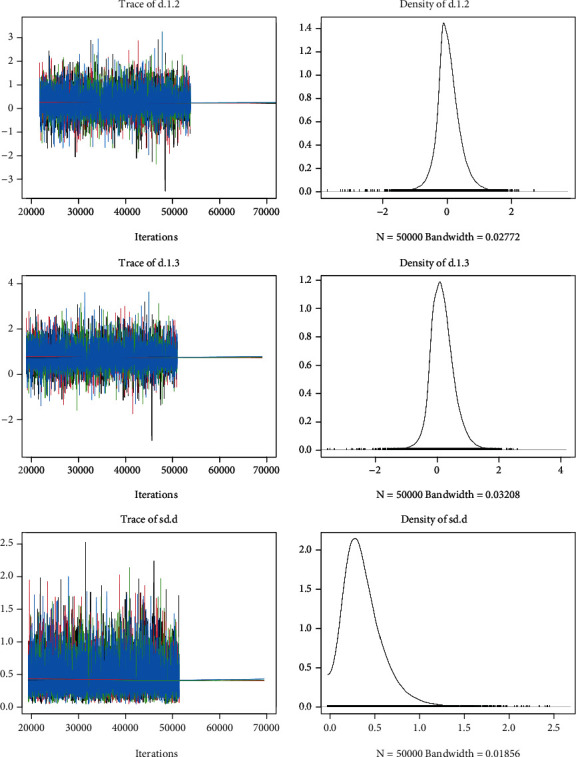
Trace and marginal density plots of OS.

16 studies provided the number of pelvic and para-aortic lymph nodes removed by different surgical approaches. Our study showed that there is no significant difference in the number of pelvic or para-aortic lymph nodes removed among RAS, LS, and LT. Results are shown in Figures 3(e) and 4(f) (*p* > .05).

14 studies reported the total (pelvic and para-aortic) number of lymph nodes directly. Our study showed that there is no significant difference in the number of total lymph nodes removed among RAS, LS, and LT. Results are shown in Figure 3(g) (*p* > .05).

### 3.8. Intraoperative/Postoperative/Total Complications

23 studies reported intraoperative complications during different surgical approaches. Statistical results showed that no significant difference in the intraoperative complications among RAS, LS, and LT. Results are shown in Figure 3(h) (*p* > .05).

25 studies reported postoperative complications with different surgical approaches. Statistical results showed that the incidence of postoperative complications was the lowest for LS followed by RAS and finally LT; the results are shown in Figure 3(i). The comparison between LS and LT (*p* < .001) was statistically significant.

29 studies reported the incidence of total (postoperative and intraoperative) complications with different surgical approaches. Statistical results showed that LS had the lowest incidence of total complications, followed by RAS, and finally LT. And the comparisons between RAS and LT (*p* = .029) and LS and LT (*p* < .001) were statistically significant. Results are shown in Figure 3(j).

### 3.9. Five-Year Overall Survival (OS)

13 studies reported five-years overall survival after different surgical approaches. Brooks–Gelman–Rubin, trace, and marginal density plots showed that the network meta-analyses converged on a solution within the 50,000 iterations after the burn-in period (Figure 4). Statistical results showed no significant difference in 5-year OS between RAS, LS, and LT. Results are shown in Figure 3(k).

### 3.10. Risk of Heterogeneity, Inconsistency and Bias

Significant heterogeneity was demonstrated in the estimated blood loss data set. We found significant heterogeneity in the study by Neshat et al. [14], perhaps due to data conversion or inconsistent estimates of blood loss had the greatest effect. No significant heterogeneity was observed in the other outcome data sets.

**Figure 5:**
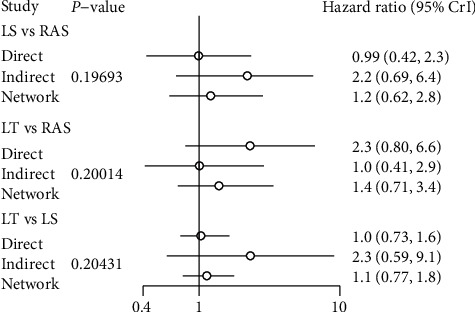
Result of node-splitting analysis for OS.

The node-splitting model showed no local inconsistency in comparisons. All node splitting inconsistency *P* values were > .05 (results of perioperative outcomes shown in Table 3 results of OS shown in Figure 5). And the loop inconsistency of perioperative outcomes also showed no inconsistency between direct and indirect comparisons (Table 3).

**Figure 6:**
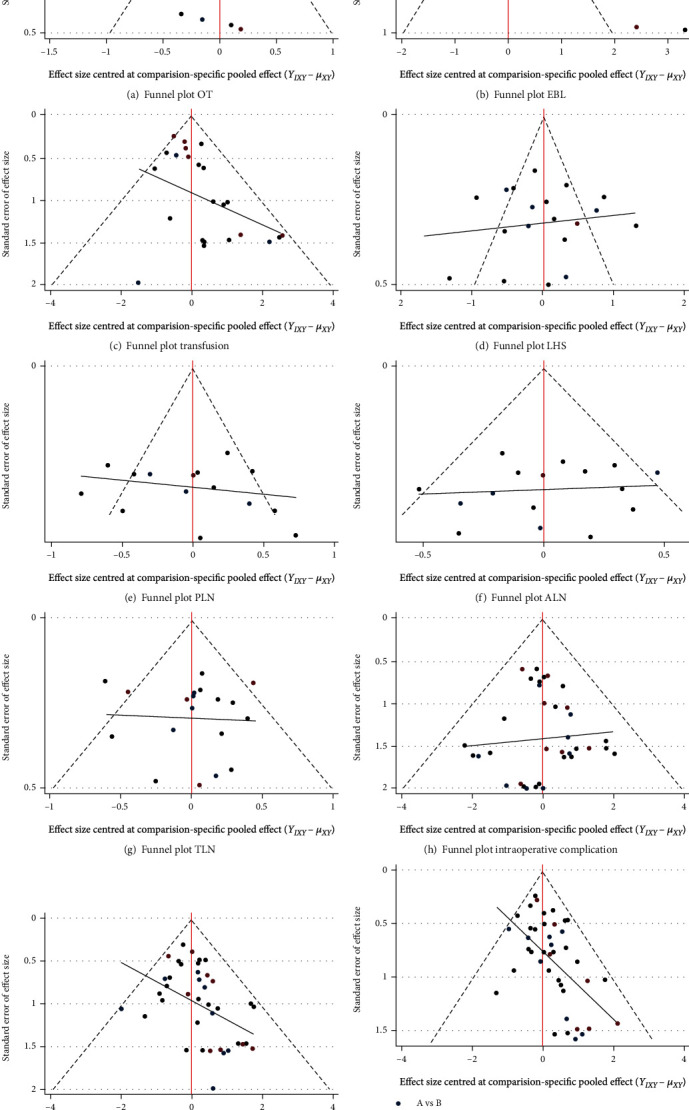
Funnel plot perioperative outcomes.

A funnel plot is used to assess the publication bias of the included literature, as shown in Figure 6. Except for pelvic lymph nodes, LHS, and EBL, the funnel plots of the other outcomes are basically symmetric, and most of the points are within the confidence interval. However, funnel plots of para-aortic lymph nodes and EBL showed certain publication bias. The risk of bias in the included literature is assessed by the NOS scale, and the score details are shown in Table 4.

## 4. Discussion

Technological advances continue to grow rapidly in the area of minimally invasive gynecologic surgery. Studies have clearly shown that minimally invasive surgery leads to faster recovery with a shorter hospital stay, improved cosmesis, decreased blood loss, and reduced postoperative pain [16]. Robotic-assisted laparoscopic surgery is the latest innovation in the field of minimally invasive surgery and is widely used in gynecologic surgery [17].

This network meta-analysis compared outcomes from 38 studies involving 8367 patients, and statistical analysis results showed no difference in the 5-year OS among the RAS, LS, and LT groups. Our analysis shows that the operating time of LT was shorter than LS (*p* = 0.02). But the beginning and end of operative time calculation were not clearly defined in the included literature. Some studies hold that RAS needs longer device preparation time than laparoscopy and laparotomy surgery [17]. For the estimated blood loss during the surgery, RAS was the least, followed by LS, and finally LT. Compared with LT, the incidence of transfusion was lower in the RAS and LS groups, and the length of hospital stay is shorter. For complications, our analysis revealed no significant differences in intraoperative complications among the three surgical approaches. However, LT had a significantly higher incidence of postoperative complications than LS. Besides, LT had a significantly higher incidence of total complications than RAS and LS. The main complications include peripheral organ damage, bleeding, deep vein thrombosis, ileus, and infection. Our analysis revealed no statistical difference for the number of pelvic/para-aortic/total lymph nodes removed by different surgical approaches.

The safety and feasibility of traditional laparoscopy and robotic-assisted laparoscopic surgery have been proved by several studies [9, 10, 18]. Minimally invasive surgery for early-stage ovarian cancer has been widely accepted, but for advanced ovarian cancer, there remains controversy. Satisfactory cytoreductive surgery is beneficial for the prognosis of patients with advanced ovarian cancer. Due to the limitations of vision and instruments using traditional laparoscopy, it is difficult to achieve satisfactory cytoreductive in advanced ovarian cancer. An observational study of stage I epithelial ovarian cancer showed that MIS was associated with an increased risk of capsular rupture, which was associated with increased mortality [19]. Besides, laparoscopy is not suitable for huge ovarian masses, and the metastasis of puncture holes needs further exploration. Some scholars still believe that prolonged midline vertical incision is the best way to perform surgery for ovarian cancer patients. Robotic-assisted laparoscopic surgery has many advantages, including but not limited to 3-dimensional view, increased dexterity, tremor filtration, and a more favorable learning curve compared with video-assisted laparoscopy [20]. RAS improves the vision and instrument limitations of traditional laparoscopy, but the disadvantages still exist, such as long preparation time, high cost, and the instruments cannot replace the sense of the operator's fingers. Maybe advanced science technology will solve this problem in the future.

The increasing trend of late childbearing has made fertility protection a problem needing attention. A multicenter cohort of 65 patients with stage I ovarian cancer undergoes fertility-sparing surgery by laparoscopy. They found that recurrence rates and survival rates in patients with ovarian cancer treated with MIS appeared to be comparable to those in patients via open surgery, and the conception rate was 60% for those women that wished to conceive after the procedure [21]. Two other similar studies have suggested that laparoscopic fertility-sparing surgery may be a viable option for patients with early EOC, but the number of cases is small, and more research is needed to explore [16, 22]. The advantages of MIS include smaller incisions and a lower risk of pelvic adhesion and inflammation, which are important for fertility protection [23].

The methodology of this network meta-analysis has potential limitations: [1] The included studies were case-control studies and cohort studies rather than randomized controlled studies. The surgeon may recommend the surgical approach to the patient based on the patient's clinical data, such as tumor size, stage, and age, which may cause particular bias. [2] The comparisons between LS and LT in the included studies are much more than that between RAS and LS or LT, and there may be a potential bias. [3] Due to insufficient literature data, we did not analyze disease-free survival (DFS) and postoperative recurrence rate. Thus, the evaluation index of the patient's postoperative prognosis is not enough, which needs more clinical research in the future.

## 5. Conclusions

In conclusion, our analysis showed that RAS and LS had a shorter hospital stay, decreased blood loss, fewer complications, and transfusion than LT. The survival outcomes of ovarian cancer patients have no difference between RAS, LS, and LT groups. There is a potential limitation of our network meta-analysis. More high-quality randomized controlled studies are needed, especially for advanced and recurrent ovarian cancer treated by robotic-assisted laparoscopic surgery. Thus, ovarian cancer patients will have more safe and effective surgical approach options.

## Figures and Tables

**Table 1:** PubMed search strategy.

#1	“Ovarian neoplasms”[mesh]
#2	(((Ovarian Neoplasm[Title/Abstract]) OR (Ovarian Cancer[Title/Abstract])) OR Ovarian Carcinoma[Title/Abstract])) OR (Ovarian Tumer[Title/Abstract])
#3	(((Peritoneoscopy[Title/Abstract]) OR (Celioscopy[Title/Abstract])) OR (Laparoscope[Title/Abstract])) OR (Endoscope[Title/Abstract])
#4	(Laparotomy[Title/Abstract]) OR (Open surgery[Title/Abstract])
#5	(((Robot-Assisted Surgery[Title/Abstract]) OR (Robot Surgery[Title/Abstract])) OR (Robot enhanced procedures[Title/Abstract])) OR (Robotic Surgical Procedure[Title/Abstract])
#6	#1 OR #2
#7	#3 OR #4 OR #5
#8	#6 AND #7

**Table 2:** Characteristics of included studies.

Study	Study year	Location	Stage	Group	N.	OS	Outcomes	Study design	Bias score	Follow up(m)
1 Chi [24] 2005	2000-2003	USA	I	LS	20		①②③⑥	Retrospective cohort	7	NA
				LT	30					
2 Ke-qin Hua [25] 2005	2002-2004	China	I	LS	10		③④⑤⑥⑨	Retrospective cohort	6	NA
				LT	11					
3 Ghezzi [26] 2007	1997-2003	Italy	I	LS	15		①②③④⑤⑥⑦⑧⑩	Retrospective cohort	7	4-108
				LT	19					
4 Jeong-Yeol Park [27] 2008	2004-2007	Korea	I	LS	19		①②③④⑤⑥⑦⑧⑨⑩	Prospective cohort	7	1-44
				LT	33					
5 Jeong-Yeol Park [28] 2008	2001-2006	Korea	I	LS	17		①②③④⑤⑥⑦⑧⑨⑩	Retrospective cohort	7	5-61
				LT	19					
6 Tzu-I Wu [29] 2010	1984-2006	Taiwan	I	LS	34	√	——	Retrospective cohort	8	2-276
				LT	174					
7 Magrina [30] 2011	2002-2008	USA	NA	R	25		③④⑤⑥⑦⑧	Retrospective case–control	8	1-128
				LS	27					
				LT	119					
8 Feuer [31] 2013	2008-2012	USA	I-IV	RAS	63		②③④⑤⑥⑨	Retrospective cohort	7	12
				LT	26					
9 Gremeau [32] 2014	1989-2009	France	I-IV	LS	7		②	Retrospective cohort	8	8-240
				LT	13					
10 Nezhat [15] 2014	2008-2012	USA	I	RAS	9		③④⑤⑥	Retrospective cohort	8	NA
				LS	10					
				LT	3					
11 Nezhat [15] 2014	2008-2012	USA	II-IV	RAS	10		③④⑤⑥⑩	Retrospective cohort	8	NA
				LS	29					
				LT	8					
12 Bogani [33] 2014	2003-2010	Italy	I-III	LS	35	√	①②③④⑤⑥⑦⑧⑩	Retrospective cohort	8	37-278
				LT	32					
13 Liu [34] 2014	2002-2010	China	I-II	LS	35		②③⑨	Retrospective cohort	8	36-84
				LT	40					
14 Zhang [35] 2014	2010-2013	China	I-III	LS	15	**√**	②③⑤⑥⑨	Retrospective cohort	6	NA
				LT	20					
15 Yu-Jin Koo [36] 2014	2006-2012	Korea	I-II	LS	24		①②③⑦⑧	Retrospective cohort	8	>60
				LT	53					
16 Favero [37] 2015	2011-2014	Germany	IIIc–IVa	LS	10		②③⑤⑥⑩	Prospective cohort	7	34
				LT	11					
17 Chen [38] 2015	2005-2014	Taiwan	IA–IIIC	RAS	44		②③④⑤⑥⑨⑩	Retrospective cohort	7	29.6
				LS	21					
				LT	73					
18 Bellia [39] 2016	2006-2014	Italy	I-III	RAS	16		②③④⑤⑥⑦⑧	Retrospective cohort	7	4-42
				LS	23					
19 Minig [40] 2016	2006-2014	Spain/Argentina	I-IV	LS	50	√	①②③④⑤⑥⑦⑧⑩	Retrospective cohort	8	>60
				LT	58					
20 Ditto [41] 2016	2005-2015	Italy	I	LS	50	√	①②③④⑤⑥⑦⑧⑩	Retrospective cohort	8	>60
				LT	50					
21 Lu [42] 2016	2002-2014	China	I-III	LS	42	√	①②③④⑤⑥⑦⑧⑨⑩	Retrospective cohort	8	16–152
				LT	50					
22 Gallotta [43] 2016	2014-2016	Italy	I	RAS	32		①②③④⑤⑥⑦⑧⑨	Case-control	6	NA
				LS	64					
23 Gallotta [44] 2016	2000-2013	Italy	I	LS	60	√	②⑨	Retrospective cohort	7	48
				LT	120					
24 Gueli Alletti [45] 2016	2013-2014	Rome	I-IV	LS	30		①②③④⑤⑥	Retrospective case-control	7	24
				LT	65					
25 Xiong Wei [46] 2017	2007-2014	China	I-II	LS	71	√	①②③⑥⑦⑧⑩	Retrospective cohort	8	3-103
				LT	31					
26 Ye Mingxia [47] 2017	2014-2015	China	I	RAS	9		①②③④⑤⑥⑨	Retrospective cohort	8	12-24
				LS	10					
				LT	8					
27 Huamao Liang [48] 2017	2007-2016	China	II-IV	LS	64	√	③④⑤⑥⑦⑧⑩	Retrospective cohort	8	5-122
				LT	68					
28 Ceccaroni [49] 2017	2007-2015	Italy	III–IV	LS	21		②③④⑤⑥⑩	Prospective cohort	8	>100
				LT	45					
29 Melamed [50] 2017	2010-2012	USA	IIIC-IV	LS	450	√	NA	Retrospective cohort	7	60
				LT	2621					
30 Nam [51] 2017	2001-2014	Korea	I-II	LS	25	√	①③④⑤⑥⑦⑧	Retrospective cohort	8	>60
				LT	24					
31 Brown [52] 2018	2006-2017	USA	III-IV	LS	53	√	①②③⑩	Retrospective cohort	7	>100
				LT	104					
32 Bergamini [53] 2018	1965-2017	Italy	I	LS	93	√	——	Retrospective cohort	7	>200
				LT	130					
33 Chen Shuying [54] 2019	2015-2018	China	III-IV	RAS	32	√	①②③⑥	Retrospective cohort	8	7-36
				LS	30					
34 Jeremie [55] 2019	2008-2014	Canada	III–IV	RAS	57	√	①⑩	Retrospective cohort	7	>60
				LT	34					
35 Facer [56] 2019	2010-2014	USA	I	RAS	636	√	②⑨	Retrospective cohort	7	>60
				LS	1265					
36 Sang [57] 2020	2008-2017	Korea	I-IV	LS	57		③⑥⑩	Retrospective cohort	7	NA
				LT	192					
37 Baiomy [58] 2020	2016-2019	Egypt	I-III	LS	30		①②③④⑤⑥⑦⑧	Retrospective cohort	7	36
				LT	30					
38 She Yujia [59] 2020	2013-2018	China	NA	RAS	33		①③④⑤⑥⑦⑧⑨⑩	Retrospective cohort	8	8-56
				LS	52					
				LT	75					
39 Margaux Merlier [60] 2020	2000-2018	French	I-II	LS	37	√	④⑤⑥	Retrospective cohort	8	18-58
				LT	107					

Note: ① estimated blood loss: EBL/ml; ② length of hospital stay: LHS/days; ③ operating time: OT/min; ④ postoperative complication; ⑤ intraoperative complication; ⑥ total complication; ⑦ pelvic lymph nodes; ⑧ para-aortic lymph nodes; ⑨ total lymph nodes; ⑩ transfusion; OS: overall survival (five years); NA: not available.

**Table 3:** Results of node-splitting model and loop inconsistency of perioperative outcomes.

Outcome	Side	P	Tau	Loop inconsistency	
				*IF*	*CL (95%)*
OT	A B	0.46	1.02	0.32	(0.00,1.51)
	A C	0.67	1.02		
	B C	0.25	1.00		
EBL	A B	0.06	0.43	0.18	(0.00,1.03)
	A C	0.33	0.47		
	B C	0.98	0.46		
Transfusion	A B	0.30	0.00	0.45	(0.00,1.58)
	A C	0.78	0.17		
	B C	0.24	0.16		
LHS	A B	0.15	1.13	1.28	(0.00,3.03)
	A C	0.10	1.11		
	B C	0.23	1.14		
Pelvic lymph nodes	A B	0.61	0.89	0.62	(0.00,2.72)
	A C	0.50	0.88		
	B C	0.53	0.88		
Para-aortic lymph nodes	A B	0.67	1.90	0.49	(0.00,4.57)
	A C	0.79	1.91		
	B C	0.99	1.92		
Total lymph nodes	A B	0.17	0.42	0.50	(0.00,1.49)
	A C	0.06	0.39		
	B C	0.12	0.41		
Intraoperative complications	A B	0.16	0.00	0.88	(0.00,2.33)
	A C	0.14	0.00		
	B C	0.41	0.00		
Postoperative complications	A B	0.08	0.00	0.70	(0.00,2.09) |
	A C	0.31	0.33		
	B C	0.06	0.00		
Total complications	A B	0.41	0.40	0.12	(0.00,1.12)
	A C	0.61	0.41		
	B C	0.95	0.41		

**Table 4:** The NOS score of the included literature.

Study	Year	Selection				Comparability	Assessment of outcome	Follow-up	Adequacy of follow-up	Scores
		1	2	3	4					
Chi	2005	∗	∗	∗	∗	∗	∗	∗		7
Ke-qin Hua	2005	∗	∗	∗	∗	∗	∗			6
Ghezzi	2007	∗	∗	∗	∗	∗	∗	∗		7
Jeong-Yeol Park	2008	∗	∗	∗	∗	∗	∗	∗		7
Jeong-Yeol Park	2008	∗	∗	∗	∗	∗	∗	∗		7
Tzu-I Wu	2010	∗	∗	∗	∗	∗	∗	∗	∗	8
Magrina	2011	∗	∗	∗	∗	∗	∗	∗	∗	8
Feuer	2013	∗	∗	∗	∗	∗	∗	∗		7
Gremeau	2013	∗	∗	∗	∗	∗	∗	∗	∗	8
Nezhat	2014	∗	∗	∗	∗	∗	∗	∗	∗	8
Bogani	2014	∗	∗	∗	∗	∗	∗	∗	∗	8
Liu	2014	∗	∗	∗	∗	∗	∗	∗	∗	8
Zhang	2014	∗	∗	∗	∗	∗	∗			6
Yu-Jin Koo	2015	∗	∗	∗	∗	∗	∗	∗	∗	8
Favero	2015	∗	∗	∗	∗	∗	∗	∗		7
Chen	2015	∗	∗	∗	∗	∗	∗	∗		7
Bellia	2016	∗	∗	∗	∗	∗	∗	∗		7
Minig	2016	∗	∗	∗	∗	∗	∗	∗	∗	8
Ditto	2016	∗	∗	∗	∗	∗	∗	∗		7
Lu	2016	∗	∗	∗	∗	∗	∗	∗	∗	8
Gallotta	2016	∗	∗	∗	∗	∗	∗			6
Gallotta	2016	∗	∗	∗	∗	∗	∗	∗		7
Gueli Alletti	2016	∗	∗	∗	∗	∗	∗	∗		7
Xiong Wei	2017	∗	∗	∗	∗	∗	∗	∗	∗	8
Ye Mingxia	2017	∗	∗	∗	∗	∗	∗	∗	∗	8
Huamao Liang	2017	∗	∗	∗	∗	∗	∗	∗	∗	8
Ceccaroni	2017	∗	∗	∗	∗	∗	∗	∗	∗	8
Melamed	2017	∗	∗	∗	∗	∗	∗	∗		7
Nam	2017	∗	∗	∗	∗	∗	∗	∗	∗	8
Brown	2018	∗	∗	∗	∗	∗	∗	∗		7
Bergamini	2018	∗	∗	∗	∗	∗	∗	∗		7
Chen Shuying	2019	∗	∗	∗	∗	∗	∗	∗	∗	8
Jeremie	2019	∗	∗	∗	∗		∗	∗	∗	7
Facer	2019	∗	∗	∗	∗	∗	∗	∗		7
Sang	2020	∗	∗	∗	∗	∗	∗	∗		7
Baiomy	2020	∗	∗	∗	∗	∗	∗	∗		7
She Yujia	2020	∗	∗	∗	∗	∗	∗	∗	∗	8
Margaux Merlier	2020	∗	∗	∗	∗	∗	∗	∗	∗	8

## Data Availability

The data used to support the findings of this study are included within the article.
